# Bioinformatic and Experimental Analysis of T Cell Immune Reactivity to SARS-CoV-2 and its Variants

**DOI:** 10.3389/fbinf.2022.876380

**Published:** 2022-05-24

**Authors:** Alison Tarke, Alba Grifoni, Alessandro Sette

**Affiliations:** ^1^ Center for Infectious Disease and Vaccine Research, La Jolla Institute for Immunology (LJI), La Jolla, CA, United States; ^2^ Department of Internal Medicine and Department of Experimental Medicine, University of Genoa, Genoa, Italy; ^3^ Department of Medicine, Division of Infectious Diseases and Global Public Health, University of California, San Diego, San Diego, CA, United States

**Keywords:** SARS-CoV-2, T cells, variants, epitopes, vaccination

## Abstract

Definition of the T cells responses to SARS-CoV-2 and associated variants is critical to understanding the complexity of adaptive immunity against SARS-CoV-2 infection. Several groups have investigated the T cells responses by both experimental and bioinformatical approaches. Here we summarize recent findings on CD4 and CD8 T cell responses to SARS-CoV-2 with particular emphasis on emerging variants of concern, consolidating the results on the impact of SARS-CoV-2 variants on T cell responses by performing an additional metanalysis emphasizing the lower impact of variant mutations in dominant T cell epitopes. The consensus is that the majority of T cell responses are conserved across all current SARS-CoV-2 variants, including Delta and Omicron. Thus, even in concomitance with reduced antibody and B cell responses, T cells can still provide a second line of antiviral immunity.

## Introduction

Herein we present a review of studies related to T cell response against the various SARS-CoV-2 variants, including the recently described Delta and Omicron variants. Supporting this special issue are additional reviews offering complimentary perspectives on the literature including those by Kedzierska and Thomas as well as DeGrace et al., among others (DeGrace et al., 2022; Kedzierska and Thomas, 2022). In this review, we cover both the bioinformatic and experimental levels of T cell responses to SARS-CoV-2 variants, with particular emphasis on our own work. Cellular immune responses to SARS-CoV-2 variants, specifically CD4 and CD8 T cells, are investigated based on complementary assays measuring cytokine release (ELISPOT), cytokine production (ICS) or T cell activation (AIM). The T cell epitopes recognized are cataloged as numerous and broadly spanning the viral proteome, in part due to the polymorphic nature of the HLA. The results of these analyses cover both the bioinformatic and experimental levels. Overall, the bioinformatic analysis of the breadth of responses and epitope conservation inspired and guided the experimental work that characterized T cell and antibody responses, and thereby, the two approaches were found to be highly complementary and synergistic.

## Initial Experimental Analysis on Breadth of T Cell Responses to SARS-CoV-2

Several studies were performed in 2020 and 2021 to define the immunodominance and breadth of SARS-CoV-2, specifically CD4 and CD8 T cell responses (Grifoni et al., 2020; Tarke et al., 2021a; Dan et al., 2021). These studies entailed close to 100 different subjects that were analyzed in terms of the T cell responses to overlapping peptide pools spanning the spike protein and other antigens related to the entire SARS-CoV-2 proteome. The findings confirmed previous studies (Ferretti et al., 2020; Le Bert et al., 2020; Sekine et al., 2020; Nelde et al., 2021), underlining how the response of human CD4 and CD8 T cells is multi-antigenic. The antigenic breadth of the responses against SARS-CoV-2 had been pointed out in previous studies. However, a larger and more comprehensive study shows that each donor in the course of natural infection is able to recognize three to four different viral antigens (Tarke et al., 2021a).

This study further identified which specific antigens each donor responded to and therefore enabled detailed deconvolution of the responses and identification of the specific epitopes recognized. Within each viral antigen, this study revealed a large breadth of response, and it was conservatively estimated that 30 to 40 different epitopes would be recognized in each donor (15–20 HLA Class II restricted CD4 and 15–20 HLA class I restricted CD8 epitopes) (Tarke et al., 2021a). This estimate suggests that SARS-CoV-2-specific T cells recognize more epitopes per donor than what was previously observed for other RNA viruses, such as dengue where an average of 11.6 and 7 epitopes are recognized by CD4^+^ and CD8^+^ T cell epitopes, respectively (Weiskopf et al., 2013; Grifoni et al., 2017). The specific epitopes differed from one subject to the next, as expected due to the general notion that epitope recognition is HLA restricted, and HLA molecules are highly polymorphic. Thus, the large breadth of epitopes recognized at the donor level was further broadened by the HLA polymorphism commonly presented in human population, although additional immunological factors can shape the repertoire of epitopes recognized in the T cell response, including the TCR recognition (Peters et al., 2020).

## Metanalysis of Human SARS-CoV-2 Responses Reveals More Than 2,000 Different Human T Cell Epitopes

The results of the Tarke et al. study were further extended in a series of subsequent analyses in which a meta-analysis was performed on all the epitopes that have been described in the literature, not only from our studies, but also from other studies independently performed by different groups (Grifoni et al., 2021). This analysis identified a total of 1,400 human T cell epitopes that had been described in 25 different studies. As these results were obtained by querying the IEDB (www.IEDB.org) (Vita et al., 2019) in early 2021, after publication the epitope query was repeated in January 2022 and the updated total experimentally defined human T cell epitopes was over 2,000, as described in 66 different studies. In conclusion, this data demonstrates a large breadth of responses, and a sizable number of epitopes recognized in humans.

The continued increase in the number of described epitopes suggest that the actual number is likely to be much greater than what has been identified to date. The fact that the HLA alleles most frequent in common ethnicities are over represented in the available studies also suggest that the breadth of responses will be larger than currently reported.

These analyses have been fundamental in laying the foundations of our understanding of T cell immunity and SARS-CoV-2 variants. In particular, they illustrated and predicted how the large breadth of responses would make it very difficult for any particular variant to escape T cell recognition at the level of human populations, because that would require the mutation of a large number of epitopes.

## Bioinformatics and Experimental Analysis of T Cell Responses to Early SARS-CoV-2 Variants

During the course of 2021 the appearance of several SARS-CoV-2 variants were important events, adding complexity to the ongoing pandemic. Several Variants Of Concern (VOC) and Variants Of Interest (VOI) were identified. Bioinformatic analyses were performed on four VOC/VOIs (Tarke et al., 2021b), namely Alpha, Beta, Gamma and Epsilon, utilizing the 1,400 experimentally defined epitopes described above in early 2021. The conservation of CD4 and CD8 T cell epitopes was assessed in these variants, and the epitopes found throughout the genome (relevant for natural infection) or limited to only the spike antigen (relevant for vaccine-induced responses) were analyzed separately. Overall, 93 and 97% of genome-wide CD4 and CD8 T cell epitopes, respectively, were 100% conserved in these variants; 85 and 95% of spike CD4 and CD8 T cell epitopes were 100% conserved. These results were confirmed by the bioinformatic analysis of Redd and co-authors (Redd et al., 2021a), which found that previously defined epitopes (Kared et al., 2021) were conserved in the newly described variants. Additional bioinformatic analysis confirmed these findings after cataloging the specific locations of variant mutations within the proteome and concluding that a large fraction of epitopes were not affected by mutations. In fact, new mutations within these variants generated novel epitopes recognized by T cells (Altmann et al., 2021).

Subsequently, and in parallel with bioinformatic analysis, the Tarke et al. study also experimentally defined the impact of early variant of concern (alpha, beta and delta) associated mutations on T cell responses measured in terms of activation and IFNγ production from subjects that were either vaccinated or naturally infected. In vaccinated subjects, CD4 and CD8 T cell responses against the spike protein were measured, while in naturally infected individuals, the pattern of CD4 and CD8 responses against the full genome was analyzed. It was found that T cells of exposed donors or vaccinees still effectively recognized SARS-CoV-2 variants, and the majority of the T cell response was unaffected (Tarke et al., 2021b).

These studies were confirmed by additional independent results that showed largely unaffected T cell activity (Alter et al., 2021; Geers et al., 2021). Specifically, Geers and co-authors showed that while the SARS-CoV-2 VOCs partially escaped humoral immunity, the T cell responses in both COVID-19 convalescent donors and vaccinees were preserved (Geers et al., 2021). Similar conclusions were reached by G. Alter and others focusing on the Ad26. COV2 vaccine, where vaccine-induced immune responses were found to be largely conserved at the level of CD4 and CD8 T cell responses against the Delta variant (Alter et al., 2021).

Overall, the consensus of the results from both bioinformatic and experimental studies was that the majority of CD4 and CD8 T cell epitopes were 100% conserved in these variants, the T cells of exposed donors or vaccinees effectively still recognized SARS-CoV-2 variants, and the majority of the T cell response was unaffected.

It is possible that variants might be associated with the creation of novel epitopes, in instances where the mutation might create new HLA binding capacities, or positively affect TCR recognition. It is conversely possible that these novel epitopes might be less readily recognized because of imprinting of T cell responses. While of theoretical concern in the context of continued boosting, imprinting of T cell responses has not been clearly described to the best of our knowledge in the case of SARS-CoV-2.

## T Cell Reactivity is Largely Preserved in a Panel of Later-Origin Variants, Including Omicron

Later in 2021, additional variants appeared including several VOCs and VOIs, such as Kappa, Delta, Lambda, Mu, and also Omicron. In Tarke et al. (2022), the previously studied Alpha, Beta, Gamma and Iota were investigated along with these more recent SARS-CoV-2 variants. The approach taken to study these variants was, as before, to perform T cell activation assays with PBMCs from vaccinated donors and test recognition of overlapping peptides pools corresponding to the sequence of the different variants.

In this study published in January 2022 in Cell, subjects were analyzed that had been vaccinated with different COVID-19 vaccines, including Moderna mRNA-1273, Pfizer BNT162b2, J&J Ad26.COV2.S or Novavax NVX-CoV2373 (Tarke et al., 2022). T cell reactivity was measured 2 weeks after one vaccination dose or after fully vaccinated time point. Fully vaccinated here indicates 2 weeks after the second dose for mRNA vaccines and a corresponding 5–6 weeks after the Adenoviral vector vaccination. Additional experiments analyzed T cell responses in the memory phase, three and a half months after vaccination. The first set of results was derived by measuring CD4 T cell responses with the full panel of variants, including Delta. At that point in time, Omicron was not included because it had not yet been described when the experiments were performed.

The results from this panel of variants, indicated that the T cell response was largely maintained at the population level. Utilizing the Activation Induced Marker (AIM) assay, 84% of the CD4 response was maintained at the fully vaccinated stage, regardless of the vaccine platform that was considered. Similar data were obtained when the CD8 T cell response was examined in the different vaccine platforms, with preservation of 81% or more of the response against these variants. These results were confirmed by Intracellular Cytokine Secretion (ICS) assays, where IFNγ, IL-2, TNFα cytokines and Granzyme B were measured; both CD4 and CD8 responses were largely preserved, with 76% or more of the total response preserved at the population level in the different variants. In terms of polyfunctionality no differences were observed between the quality of CD4 and CD8 responses against ancestral strain as compared to the other variants.

Finally, a similar pattern was noted when the responses of different subjects were evaluated after a single immunization (2 weeks after the first or single immunization) at the level of both CD4 and CD8 T cell responses.

A subsequent set of experiments addressed responses against the Alpha, Beta, Gamma and Delta variants at three and a half months after immunization, and a similar picture was noted with 80% or more of the total CD4 and CD8 T cell response retained in the AIM assay. At the level of ICS assays, the majority of the responses were preserved; however, there were some significant decreases in the case of the Delta variant, where 80% of the CD4 response was maintained, and 61% of the CD8 response was maintained.

The impact of variant associated mutations on T cell responses was then assessed five to 6 months post vaccination. This time point was chosen as representative of an immune memory stage. In the AIM assay, 84 and 85% of the CD4 and CD8 T cell responses against Omicron were retained, respectively. In the case of the ICS assay, the response to the Omicron spike peptide pool was actually 110% of what was observed to the Ancestral spike for CD8, and 93% for CD4 T cell responses (Tarke et al., 2022).

In conclusion, T cell recognition of the different variants was largely retained in the AIM assay for both CD4 and CD8 in all vaccine platforms tested. Responses were also retained at the level of the ICS assay, thereby highlighting that the functionality and multi-functionality of responses is maintained. T cell responses were largely preserved in fully vaccinated subjects, after a single immunization, and at the 3.5 and 6 months memory time point. These results confirmed the findings obtained at the earlier time points, which demonstrated that a majority of the reactivity was effectively preserved for both CD4 and CD8 T cells, regardless of the variants analyzed, and in particular effective recognition was still preserved for the Omicron variant.

## Bioinformatic Analysis of Conservation of T Cell Epitopes in a Large Panel of SARS-CoV-2 Variants

In the next series of analyses, to gain more insight into the mechanisms by which preservation of a large fraction of the T cell response was achieved we combined the information related to experimentally defined epitopes available in IEDB (www.IEDB.org) and the list of amino acid mutations per each variant. In the first set of analyses, the fraction of epitopes that was 100% conserved in early variants was compared to those in the late variants. Early variants were considered to be the ones previously analyzed (Tarke et al., 2021b), namely Alpha, Beta, Gamma, and Epsilon, while the later variants included Kappa, Delta, Lambda, and several others. The rationale for comparing how many CD4 and CD8 T cell epitopes would be 100% conserved was to address whether the continued evolution of the variants would be associated with an increased fraction of mutated epitopes, possibly suggestive of a continued and progressive trend towards escape from T cell recognition.

The results of the analysis did not support the notion of a continued increase in mutations. Specifically, when the epitopes derived from the full proteome, as relevant in the context of natural infection, were analyzed to compare early versus late variants, 94% of the CD4 epitopes were 100% conserved in early variants as opposed to 97% of the T cell epitopes that were conserved in late variants. In the case of CD8 epitopes in the full proteome, 89% were totally conserved in early variants, while in the later variants, 95% of the CD8 epitopes were totally conserved. Similar results were observed if the analysis was limited to only the spike protein, which is relevant for vaccination.

In the case of the Omicron variant, which is associated with a higher number of mutations as compared to the other variants analyzed, it would be expected to find a higher number of T cell epitopes containing mutations. Indeed, there was a significant increase in the number of epitopes that were mutated. However, the majority of the T cell epitopes were still 100% conserved; in the case of spike, 72% of the CD4 T cell epitopes and 86% of the CD8 T cell epitopes were completely conserved in Omicron.

Furthermore, it should be noted that these analyses refer to the number of T cell epitopes that are 100% conserved in the various variants and not necessarily an indication of the level of preservation of T cell responses at the functional level. Even if an epitope is mutated within a specific variant, the epitope could still be effectively cross recognized by T cells. Consistent with this notion, 70%–75% of the mutated epitopes were predicted to still bind effectively to the relevant HLA class I molecules using the recommended algorithm for class I prediction in IEDB (Dhanda et al., 2019). The analyses described herein have not considered the conservation as a function of the HLA supertype. This would be an interest topic for further investigations.

In conclusion, the majority of T cell epitopes are 100% conserved in all variants analyzed, particularly the S2 region of spike, and of the minority of epitopes that are mutated a large fraction may actually be functionally recognized by T cells. It is worth to note that those conclusions are limited by the fact that this analysis was carried on a small number of VOCs without a comprehensive survey in a phylogenetic context.

## Predicting the Impact of Omicron Mutations in T Cell Epitopes

**FIGURE 1:**
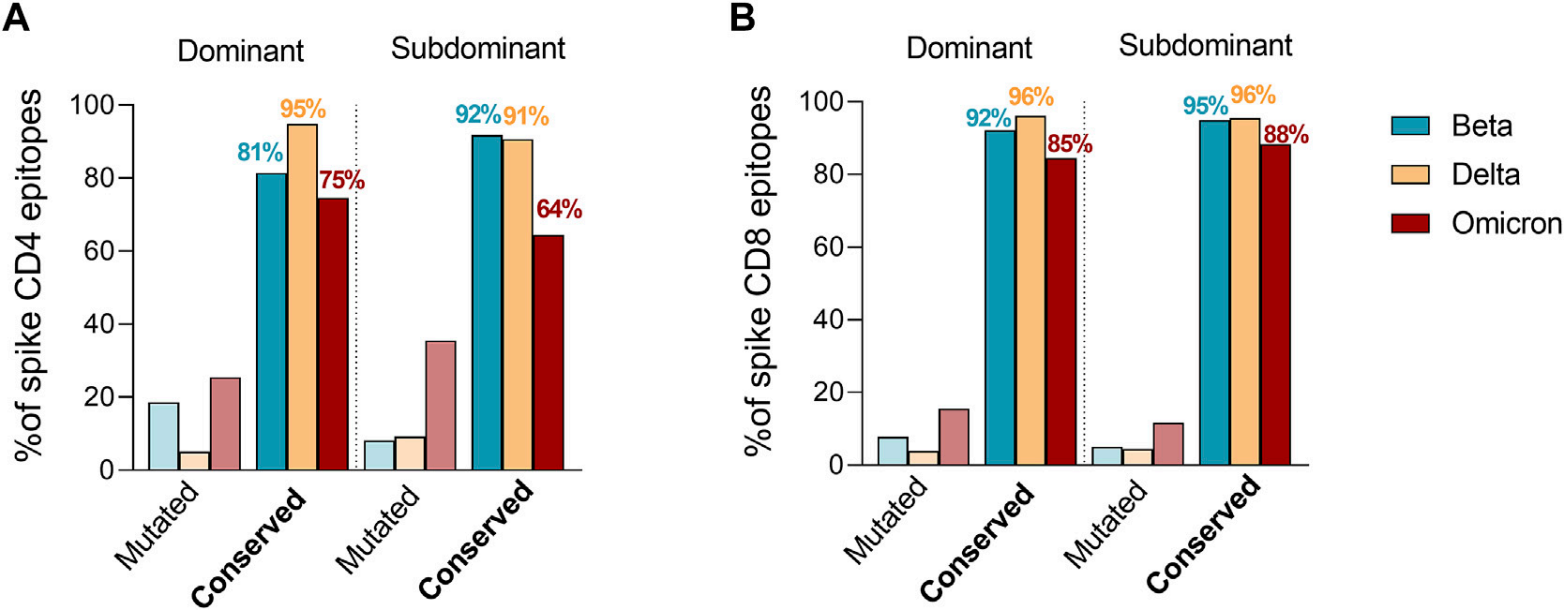
Effect of Beta, Delta and Omicron spike mutations on T cell epitope immunodominance. Percentage of ancestral SARS-CoV-2 CD4^+^ **(A)** and CD8^+^ **(B)** T cell epitope sequences affected by Beta (green), Delta (yellow) and Omicron (red) variants. The epitope list was extracted from IEDB (www.IEDB.org) and divided based on a frequency of responders greater or equal than 3 (dominant) or less then 3 (subdominant). Percentages of conserved epitopes are noted per each SARS-CoV-2 variant separately.

Tarke et al. then investigated whether Omicron mutations may occur more frequently in epitopes, as compared to the rest of the spike sequence, which would be indicative of Omicron mutations being selected to facilitate escape from T cell recognition. The analysis revealed that 82% of the full set of nine-mers encompassing the entire spike proteins were still conserved in Omicron, compared to 86% of CD8 epitopes. Therefore, there is no indication that Omicron mutations are selectively enriched in T cell epitopes. To exclude the idea that Omicron mutations were preferentially impacting more dominant epitopes, which would also be suggestive of immune pressure at the level of T cell epitopes, T cell epitopes available in IEDB were subsequently classified as dominant, as being positive in three or more donors, and subdominant epitopes, as being recognized at a lower frequency as previously reported (Yu et al., 2022). These criteria were based on a meta-analysis of the data curated in the IEDB. We clarify that the IEDB does not curate response magnitude data, and curates published data based on response frequencies, including all author’s reported assay methodologies, including activation, cytokine production tetramer staining, ELISPOT and others. The selection of epitopes positive in at least three donors ensures that the epitopes are broadly recognized in multiple subjects. Raising the cutoff further, would results in biased representation in epitopes restricted by the most frequent alleles. Based on dominant and subdominant epitope classification, the number of mutated epitopes were then compared to fully conserved epitopes considering the SARS-CoV-2 variants showing the most detrimental impact in the immune response (Figure 1). The fraction of dominant (D) and subdominant (SD) mutated spike epitopes was overall comparable with different trends of conservation. Specifically, a higher conservation frequency in dominant epitopes was observed for Delta for CD4 (D = 95%; SD = 91%) and no differences for CD8 (D = 96%; SD = 96%). Omicron had a similar trend to Delta for the CD4 epitopes (D = 75%; SD = 64%) while showing an inverted trend in the context of the CD8 epitopes (D = 85%; SD = 88%). Beta showed an inverted trend with higher conservancy in subdominant epitopes for both CD4 (D = 81%; SD = 92%) and CD8 (D = 92%; SD = 95%) epitopes. Overall, 75 and 85% or more of D epitopes for CD4 and CD8, respectively, were conserved in any variant considered.

In conclusion, taking together the bioinformatics analyses described herein and considering both CD4 and CD8 T cell epitopes related to the entire SARS-CoV-2 proteome or only the spike protein and also considering the amino acid mutation contained in the panel of variants analyzed, including Omicron, the majority of epitopes were 100% conserved in these different variants. These findings support previously published bioinformatic analyses which showed the tally of spike T cell epitopes mutated in Omicron, or provided information about previously defined epitopes conserved in Omicron (Redd et al., 2021b; Bernasconi et al., 2021; Kared et al., 2021).

Additionally, the minority of the epitopes that were mutated were predicted to still have similar binding affinity (within a 3-fold change corresponding to a range of 0.01–1 percentile rank), and those mutations were not enriched in epitopes compared to the rest of the spike protein or the non-spike proteins, or when comparing dominant and subdominant epitopes. Thus, these data do not support the notion of a selective pressure for mutation of the T cell epitopes at the population level. It is worth noting that while this has been confirmed at the experimental level *ex-vivo*, additional unknown factors may influence the expansion of specific T cell clones among the epitope pools considered herein and found to be more affected by viral mutations (Le Bert et al., 2022).

**FIGURE 2:**
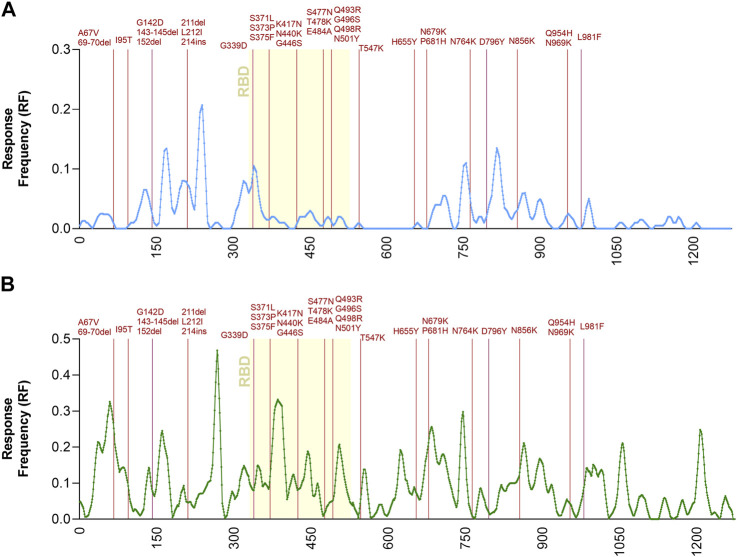
Immunodominant antigenic regions impacted by Omicron mutations. Response Frequency defined as the number of individuals and assays reporting positive responses to a peptide including that particular residue was calculated in a dataset previously reported (Grifoni et al., 2021). Only the spike protein was considered for this specific analysis. Panel **(A)** shows CD4 (light blue) while panel **(B)** shows CD8 (green) T cell epitopes. The receptor binding domain (RBD) region of the spike protein, is indicated in yellow because it is critically recognized by neutralizing antibodies and implicated in viral cell entry. The spike mutations found in Omicron related to the Ancestral sequences are noted in dark red.

To further address the relationship between T cell immunodominance and Omicron mutations, the Immunobrowser tool provided by the IEDB (Dhanda et al., 2018) was utilized. This tool extracts the information related to immune recognition and plots along the sequence of an antigen of interest the local density of recognition, compiled across different assays, donors and independently published reports. Here, positive human T cell results were selected and separately plotted for CD4 and CD8 T cell responses, following the strategy described by the recent metanalysis (Grifoni et al., 2021). On these plots the location of the Omicron-associated mutations was overlaid (Figure 2). The results visualize the regions along the spike sequence that are immunodominant for human CD4 and CD8 T cell responses. They further illustrate and demonstrate that the mutations are randomly distributed along the sequence and do not selectively cluster in T cell immunodominant regions.

## Understanding the Impact of SARS-CoV-2 Variants at the Single Donor Level

Based on the bioinformatic analyses presented above, Tarke et al. further experimentally addressed the mechanisms involved in the preservation of T cell responses against the various variants, and in particular related to the individual patterns of immunodominance. For this purpose, vaccinated donors collected 6 months after full vaccination were utilized that had undergone apheresis, and for which a significant number of cells was available to detail the epitope repertoire recognized in each different donor. For both CD4 and CD8 responses, each donor recognized a median of about 10 different CD4 spike epitopes and 10 different CD8 spike epitopes, thus confirming the large breadth of epitopes at the individual donor level (Tarke et al., 2022). This is in line with the 30 to 40 different epitopes we previously estimated to be recognized in naturally infected individuals, given that Spike accounts for 25%–30% of the total T cell reactivity at the protein level (Tarke et al., 2021a). The experiments also demonstrated that the epitope repertoire recognized by the different donors is largely non-overlapping.

Significant variation was however detected in the number of epitopes recognized by each donor, with a range of 5–43 different epitopes recognized in the various donors. This observation might help explain why at the experimental level occasional donors are associated with significant reduction in T cell reactivity, while T cell reactivity is largely preserved at the population level.

Finally, while some of the epitopes recognized in a particular donor may be mutated in one variant or another, still the majority of the epitopes was not mutated, and in fact, the majority of the overall response would still be conserved in the different variants of concern. While the calculated percentage of the responses associated with conserved epitopes was in some cases somewhat decreased (but still 65% or more was preserved), the median conservation of the response at the level of a repertoire analysis was 85% for CD8 and 80% for CD4 T cell epitopes (Tarke et al., 2022). Additionally, the mutations were not specifically found in immunodominant epitopes, but affected a broad range of epitopes depending on donor and variant analyzed, in line with the fact that epitope repertoire varies from one individual to the other. Therefore no clear viral selective pressure could be observed.

In conclusion, while some epitopes were mutated, non-mutated epitopes still accounted for 65%–100% of the response. Additionally, the T cell epitope repertoire is broad, and not associated with strong immunodominance. This data illustrates the mechanisms behind the underlying effective preservation of this recognition of Omicron.

## Emerging Consensus on Preservation of T Cell Responses Against Variants

Several studies in the last few months have addressed the issue of preservation of T cell responses against variants. The emerging consensus of several studies performed by different groups in different geographical locations and involving different patient cohorts, is that T cell responses are largely preserved in SARS-CoV-2 variants, including Delta and Omicron.

Keeton et al. (2021) reported effective recognition for CD4 and CD8 T cell responses based on polyfunctional profiles of cytokines (IFNγ, IL-2 and TNFα) measured by ICS of both Beta and Delta, even though some decrease for Delta was observed in the case of CD8 responses in cohorts of South African subjects. Melo-Gonzalez et al. (2021) highlighted how CoronaVac vaccination of Chilean subjects induced T cells that also cross-recognized different variants of concerns, including Alpha, Beta, Gamma, and Delta.

Further studies addressed T cell responses to the Omicron variant. Studies of Swedish cohorts (Gao et al., 2022) defined responses associated with vaccination or natural infection with the ancestral sequence and reported preservation of a large majority of T cell responses both at the CD4 and CD8 level, and further defined the phenotype of the T cells associated with cross-recognition. Keeton et al. (2022) examined spike T cell responses induced by either vaccination or infection in South African cohorts, and also noted that a large fraction of T cell responses was preserved against Delta, Beta and Omicron. This study also detailed T cell responses from Omicron infected hospitalized patients, which were comparable to those seen in patients who were hospitalized prior to Omicron and presumably infected with Beta or Delta.

A study in the Netherlands (GeurtsvanKessel et al., 2022) focused on Omicron CD4 T cell responses after vaccination with different vaccine platforms. Again, while there were differences in magnitude and kinetic of responses induced by the various vaccine platforms, a large majority of the T cell responses in the vaccinated individuals still effectively cross-recognized the Omicron variant. A study from Madelon et al. (2021), examined Omicron recognition by T cells in anti-CD20 treated multiple sclerosis patients. Robust T cell responses against the S antigen were detected recognizing both Delta and Omicron variants. These responses were increased by the third dose of vaccination, even though some of the response against these variants was decreased in terms of magnitude, as compared to the responses observed against the ancestral spike. Additional studies also confirming highly cross-reactive and preserved CD4 and CD8 responses were reported by Liu et al., and also by Lorenzo DeMarco and coworkers (De Marco et al., 2021; Liu et al., 2022).

## Conclusion

The studies and experiments reviewed herein highlight how CD4 and CD8 T cell responses are largely preserved in vaccinated subjects for different variants, including Delta and Omicron. This was observed in the case of mRNA platforms (mRNA-1273 and BNT162b2), adenovirus-based platforms (Ad26.COV2.S and Vaxzevria), protein-recombinant platforms (NVX-CoV2373) and inactivated virus strategies (CoronaVac). Those findings were observed both at early (soon after one immunization) and memory time points, and utilizing different assay platforms (ELISPOT, AIM and ICS assays). These conclusions emerged from different studies located in different geographical locations and assessed cohorts of different ethnicity and different viral exposure and vaccination schedule profiling. Bioinformatics analyses played a pivotal role in illuminating the mechanisms of this preservation, based on a large epitope repertoire, where the majority of the T cell epitopes are fully conserved. Further detailed analysis did not provide evidence of selective accumulation of mutation in T cell epitopes, or T cell immune pressure at the population level driving variant evolution. The study of SARS-CoV-2 variants further illustrated the value of an iterative approach where bioinformatics analysis informs experimental work, and the results of experimentation guide further bioinformatic analysis.

In conclusion, while a large body of evidence demonstrated that variant associated mutations have been generally found to significantly impact antibody neutralization, CD4 and CD8 T cell recognition is largely preserved (DeGrace et al., 2022). It is hypothesized that neutralizing antibodies are the main mechanism mediating protection from infection, while T cell play a prominent role in protection from severe disease (Chen and John Wherry, 2020; Niessl et al., 2021; Sette and Crotty, 2021; Moss, 2022). Accordingly, this is consistent with the observed decreased efficacy of vaccination and natural infection in terms of protection against reinfection and breakthrough infections, but the continued efficacy observed in terms of protection from severe disease, hospitalization and death (Danza et al., 2022; Madhi et al., 2022; Maslo et al., 2022). These correlations should however be interpreted with caution, since additional factors are also likely to come into play, some related to the intrinsic difference across variants in binding affinity between the spike protein and the ACE2 receptor (Starr et al., 2020; Miotto et al., 2022), and increases in viral infectiveness (Rabaan et al., 2021), but decreased capacity to infect lung cells (Leung, 2021), all factors that could possible influence the immune response. Additional factors are also related to other immunological factors including a role for non-neutralizing antibodies (Lu et al., 2018; Webb et al., 2021), memory B cells (Moriyama et al., 2020; Roltgen and Boyd, 2021; Sette and Crotty, 2021). All the above mentioned factors could contribute to the increased susceptibility to infection and preserved protection against severe disease. The various cohorts reviewed in the manuscript were not selected on the basis of presence of specific HLA alleles, and thereby represent a snapshot of recognition at the level of the HLA alleles commonly encountered in the various populations. In that respect, it is encouraging that similar results have been noted in studies performed in different continents and widely divergent patient populations. At the same time, continued testing in a broad spectrum of HLA types, representative of worldwide ethnicities should be considered an important goal.
